# Soft *C*-continuity and soft almost *C*-continuity between soft topological spaces

**DOI:** 10.1016/j.heliyon.2023.e16363

**Published:** 2023-05-18

**Authors:** Samer Al Ghour

**Affiliations:** Department of Mathematics and Statistics, Jordan University of Science and Technology, Irbid 22110, Jordan

**Keywords:** Almost continuous functions, *c*-continuous functions, Almost *c*-continuous functions, Soft continuous functions, Soft almost continuous functions, Soft compact, Soft Hausdorff

## Abstract

We present the notions of soft *c*-continuity and soft nearly *c*-continuity, which are weaker versions of soft continuity and soft almost continuity, respectively. We obtain several characterizations for these two concepts. We show that soft *c*-continuity and soft almost *c*-continuity are preserved under soft restrictions. In addition, we investigate the conditions under which the composition of two soft *c*-continuous and soft almost *c*-continuous functions is soft *c*-continuous and soft almost *c*-continuous. Moreover, via soft N-open sets, we give a characterization of the soft compactness of soft topological spaces over finite sets of parameters. In addition, we show that for a given soft topological space (L,Ω,F), the collection of soft regular open sets with soft compact complements forms a soft base for some coarser soft topology. We demonstrate that $\left(L,\Omega^{⁎},F\right)$ are all soft compacts. Furthermore, we demonstrate that $\Omega =\Omega^{⁎}$ if (L,Ω,F) or $\left(L,\Omega^{⁎},F\right)$ is soft compact and soft Hausdorff. Finally, we investigate the correspondences between the novel notions in soft topology and their general topological analogs.

## 1 Introduction and preliminaries

Daily life presents us with a number of ambiguous problems that we are unable to resolve using traditional mathematical methods. It was suggested that scenarios such as fuzzy sets and soft sets be used to deal with these types of problems. The concept of “soft sets” was first proposed by Molodtsov [1] in 1999 as a fresh mathematical approach for dealing with confusing situations and imprecise data. As described by Molodtsov, soft sets have advantages over fuzzy sets in a number of different disciplines.

The soft sets have been used by numerous academics and researchers who are interested in uncertainty in both theoretical and applied concerns. In order to solve decision-making issues, Maji et al. [2] used soft sets in 2002, and they [3] gave the idea for a set of operations between soft sets in 2003. According to the published literature, some of these operations had shortcomings that led certain authors to change their definitions and embrace new sorts of them for a variety of reasons [4], [5], [6].

In order to build a soft topology over a specified set of parameters, Shabir and Naz [7] defined a soft topology over a family of soft sets. Soft topological concepts were established by Shabir and Naz to be equivalent to their counterparts in classical topology, which motivated and supported researchers in the field to continue in this direction. Numerous contributions have been made to the exploration of topological notions and concepts in soft environments since the development of soft topology [8], [9], [10], [11], [12], [13], [14], [15], [16], [17], [18], [19], [20], [21], [22], [23], [24], [25], [26], [27], [28], [29], [30].

Soft topology and other fields of mathematics have seen extensive research on the soft continuity. The importance of soft continuity lies in the majority of its applications in soft topological models, data modeling, engineering, science, economics, and business, among others, make it an important topic that has received a lot of attention from many scientists.

In this paper, we introduce the concepts of soft *c*-continuity and soft almost *c*-continuity which are weaker forms of soft continuity and soft almost continuity, respectively. We obtain several characterizations for these two concepts. We show that soft *c*-continuity and soft almost *c*-continuity are preserved under soft restrictions. In addition, we investigate the conditions under which the composition of two soft *c*-continuous and soft almost *c*-continuous functions is soft *c*-continuous and soft almost *c*-continuous. Moreover, via soft N-open sets, we give a characterization of the soft compactness of soft topological spaces over finite sets of parameters. In addition, we show that for a given soft topological space (L,Ω,F), the collection of soft regular open sets with soft compact complements forms a soft base for some coarser soft topology. We demonstrate that $\left(L,\Omega^{⁎},F\right)$ are all soft compacts. Furthermore, we demonstrate that $\Omega =\Omega^{⁎}$ if (L,Ω,F) or $\left(L,\Omega^{⁎},F\right)$ is soft compact and soft Hausdorff. Finally, we investigate the correspondences between the novel notions in soft topology and their general topological analogs.

Topological space and soft topological space (STS and TS, respectively) will be employed concurrently in the present research. The notions and terminology from [31], [32] will be used throughout this research.

Let (L,ς) and (L,Ω,F) be a TS and a STS, respectively. Let W⊆L and K∈SS(L,F). In this research, the terms $Int_{\varsigma }(W)$, $Cl_{\varsigma }(W)$, $Int_{\Omega }(K)$, and $Cl_{\Omega }(K)$, respectively, will be used to refer to the interior of *W* in (L,ς), the closure of *W* in (L,ς), the soft interior of *K* in (L,Ω,F), and the soft closure of *K* in (L,Ω,F); Comp(L,ς) stands for the family of all compact subsets of (L,ς), while $\varsigma^{c}$ and $\Omega^{c}$ stand for the family of closed sets on (L,ς) and the family of soft closed sets on (L,Ω,F), respectively.

Now we will review some of the notions that will be used in the continuation of the paper.

Definition 1.1 [33] Let (L,ς) be a TS and W⊆L. Then *W* is said to be a regular open set in (L,ς) if $W=Int_{\varsigma }\left(Cl_{\varsigma }\left(W\right)\right)$. The complement of a regular open set in (L,ς) is said to be a regular closed set in (L,ς). RO(L,ς) (resp. RC(L,ς)) denotes the collection of regular open sets (resp. regular closed sets) in (L,ς).

Definition 1.2 Let p:(L,ς)⟶(N,δ) be a function between TSs and let l∈L. Then

(1) [34] *p* is called almost continuous at *l* if for any W∈δ such that p(l)∈W, we find T∈ς such that l∈T and $p(T)\subseteq Int_{\delta }\left(Cl_{\delta }\left(W\right)\right)$. *p* is called almost continuous if *p* is almost continuous at each point s∈L.

(2) [35] *p* is called *c*-continuous at *l* if for any W∈δ such that p(l)∈W and N−W∈Comp(N,δ), we find T∈ς such that l∈T and p(T)⊆W. *p* is called *c*-continuous if *p* is *c*-continuous at each point s∈L.

(3) [36] *p* is called almost *c*-continuous at *l* if for any W∈δ such that p(l)∈W and N−W∈Comp(N,δ), we find T∈ς such that l∈T and $p(T)\subseteq Int_{\delta }\left(Cl_{\delta }\left(W\right)\right)$. *p* is called almost *c*-continuous if *p* is almost *c*-continuous at each point s∈L.

Definition 1.3 A soft set H∈SS(L,F) defined by

(1) [31] $H(f)=\left\{ \begin{array}{cc} Y\; & \text{if }f=d\\ \emptyset \; & \text{if }f\neq d \end{array}\right.$ is denoted by $d_{Y}$.

(2) [37] H(f)=Y for all f∈F is denoted by $C_{Y}$.

(3) [38] $H(f)=\left\{ \begin{array}{cc} \left\{l\right\}\; & \text{if }f=d\\ \emptyset \; & \text{if }f\neq d \end{array}\right.$ is denoted by $d_{l}$ and is said to be a soft point. SP(L,F) denotes the set of soft points in SS(L,F). If $a_{x},b_{y}\in SP(L,F)$, then we say that $a_{x}$ and $b_{y}$ are distinct if a≠b or x≠y.

Definition 1.4 [38] Let H∈SS(L,F) and $f_{l}\in SP(L,F)$. Then $f_{l}$ is said to belong to *H* (notation: $f_{l}\overset{˜}{\in }H$) if l∈H(f).

Definition 1.5 Let (L,Ω,F) be a STS and let M∈SS(L,F). Then

(a) [39] *M* is called a soft compact subset of (L,Ω,F) if for any A⊆Ωwith $M\overset{˜}{\subseteq }{\overset{˜}{\cup }}_{A\in A}A$, we find a finite subcollection $A_{1}\subseteq A$ such that $M\overset{˜}{\subseteq }{\overset{˜}{\cup }}_{A\in A_{1}}A$. The family of all soft compact subsets of (L,Ω,F) will be denoted by Comp(L,Ω,F). (L,Ω,F) is called soft compact if $1_{F}\in Comp\left(L,\Omega ,F\right)$.

(b) [40] *M* is said to be a soft regular open set in (L,Ω,F) if $M=Int_{\Omega }\left(Cl_{\Omega }\left(M\right)\right)$. The soft complement of a soft regular open set in (L,Ω,F) is called a regular closed set in (L,Ω,F). RC(L,Ω,F) (resp. RO(L,Ω,F) denotes the collection of soft regular closed sets (resp. soft regular open sets) in (L,Ω,F).

(c) [41] (L,Ω,F) is called soft Hausdorff if for every $a_{r},b_{s}\in SP(L,F)$ such that $a_{r}\neq b_{s}$, there exists M,N∈Ω such that $a_{r}\overset{˜}{\in }M$, $b_{s}\overset{˜}{\in }N$, and $M\overset{˜}{\cap }N=0_{F}$.

Definition 1.6 [38] Let G∈SS(L,F). Then *G* is called a finite soft set if for all f∈F, the set G(f) is finite. The collection of all finite soft sets from SS(L,F) will be denoted by FSS(L,F).

Definition 1.7 [42] Let (L,Ω,F) be a STS and let H∈SS(L,F). Then *H* is called soft N-open set in (L,Ω,F) if for each $f_{l}\overset{˜}{\in }H$, there exists K∈Ω such that $f_{l}\overset{˜}{\in }K$ and K−H∈FSS(L,F). $\Omega_{N}$ will represent the collection of soft N-open sets in (L,Ω,F).

For a given STS (L,Ω,F), it is proved in [42] that $\left(L,\Omega_{N},F\right)$ is a STS such that $\Omega_{N}$ is finer than Ω.

Theorem 1.8 *[31]* *For any TS* (L,ς) *, the family*

{H∈SS(L,F):H(f)∈ςfor allf∈F}

*is a soft topology on L relative to F. This soft topology will be denoted by* τ(ς) *.*

Theorem 1.9 *[31]* *For any collection of TSs* $\left\{\left(L,\lambda_{f}\right):f\in F\right\}$ *, the family*

$$\left\{H\in SS\left(L,F\right):H\left(f\right)\in \lambda_{f}\;\text{for all}\;f\in F\right\}$$

*is denoted by* ${⊕}_{f\in F}\lambda_{f}$ *and forms a soft topology on L relative to F.*

Definition 1.10 [43] Let $f_{pu}:\left(R,\Pi ,A\right)⟶\left(L,\Omega ,F\right)$ be a soft function and let $a_{r}\in SP(R,A)$. Then

(a) $f_{pu}$ is called soft almost continuous at $a_{r}$ if for every M∈Ω such that $f_{pu}\left(a_{r}\right)\overset{˜}{\in }M$, we find K∈Π such that $a_{r}\overset{˜}{\in }K$ and $f_{pu}\left(K\right)\overset{˜}{\subseteq }Int_{\Omega }\left(Cl_{\Omega }\left(M\right)\right)$.

(b) $f_{pu}$ is called soft almost continuous if $f_{pu}$ is soft almost continuous at each member of SP(R,A).

## 2 Soft C-continuous functions

We propose soft *c*-continuity as a novel category of soft functions and investigate some of its features. We characterize it in different ways and show that this category of soft functions strictly contains the category of soft continuous functions. We show that soft *c*-continuity is preserved under soft restrictions. In addition, we investigate the conditions under which the composite of two soft c-continuous functions is soft c-continuous. Furthermore, we characterize the soft compactness of soft topological spaces over finite sets of parameters using soft N-open sets. We also look at the connections between this family of soft functions and their general topological alternatives.

Definition 2.1 Let $f_{pu}:\left(R,\Pi ,B\right)⟶\left(L,\Omega ,F\right)$ be a soft function and $b_{r}\in SP(R,B)$. Then:

(a) $f_{pu}$ is called soft *c*-continuous at $b_{r}$ if for every M∈Ω such that $f_{pu}\left(b_{r}\right)\overset{˜}{\in }M$ and $1_{F}-M\in Comp\left(L,\Omega ,F\right)$, there exists K∈Π such that $b_{r}\overset{˜}{\in }K$ and $f_{pu}\left(K\right)\overset{˜}{\subseteq }M$.

(b) $f_{pu}$ is called soft *c*-continuous if $f_{pu}$ is soft *c*-continuous at all members of SP(R,B).

Theorem 2.2 *The following are equivalent for the soft function* $f_{pu}:\left(R,\Pi ,B\right)⟶\left(L,\Omega ,F\right)$ *:*

*(a)* $f_{pu}$ *is soft c-continuous.*

*(b) For every* M∈Ω *such that* $1_{F}-M\in Comp\left(L,\Omega ,F\right)$ *,* $f_{pu}^{-1}(M)\in \Pi$ *.*

*(c) For every* $M\in \Omega^{c}\cap Comp\left(L,\Omega ,F\right)$ *,* $f_{pu}^{-1}(M)\in \Pi^{c}$ *.*

Proof (a) ⟶ (b): Let M∈Ω such that $1_{F}-M\in Comp\left(L,\Omega ,F\right)$. Let $b_{r}\overset{˜}{\in }f_{pu}^{-1}(M)$. Then $f_{pu}\left(b_{r}\right)\overset{˜}{\in }M$. Since $f_{pu}$ is soft *c*-continuous at $b_{r}$, then we find S∈Π such that $b_{r}\overset{˜}{\in }S$ and $f_{pu}\left(S\right)\overset{˜}{\subseteq }M$. Thus, $b_{r}\overset{˜}{\in }S\overset{˜}{\subseteq }f_{pu}^{-1}(M)$. Hence, $f_{pu}^{-1}(M)\in \Pi$.

(b) ⟶ (c): Let $M\in \Omega^{c}\cap Comp\left(L,\Omega ,F\right)$. Then we have $1_{F}-M\in \Omega$ such that $1_{F}-\left(1_{F}-M\right)=M\in Comp\left(L,\Omega ,F\right)$. So, by (b), $f_{pu}^{-1}(1_{F}-M)=1_{B}-f_{pu}^{-1}(M)\in \Pi$. Hence, $f_{pu}^{-1}(M)\in \Pi^{c}$.

(c) ⟶ (a): Let $b_{r}\in SP(R,B)$ and let M∈Ω such that $f_{pu}\left(b_{r}\right)\overset{˜}{\in }M$ and $1_{F}-M\in Comp\left(L,\Omega ,F\right)$. Then we have $1_{F}-M\in \Omega^{c}\cap Comp\left(L,\Omega ,F\right)$, and by (c), $f_{pu}^{-1}\left(1_{F}-M\right)=1_{B}-f_{pu}^{-1}(M)\in \Pi^{c}$. Put $S=f_{pu}^{-1}(M)$. Then S∈Π such that $b_{r}\overset{˜}{\in }S$ and $f_{pu}\left(S\right)\overset{˜}{\subseteq }M$. Hence, $f_{pu}$ is soft *c*-continuous. □

Corollary 2.3 *Let* $f_{pu}:\left(R,\Pi ,A\right)⟶\left(L,\Omega ,F\right)$ *be a soft function such that* (L,Ω,F) *is soft Hausdorff. Then the following are equivalent:*

*(a)* $f_{pu}$ *is soft c-continuous.*

*(b) For every* M∈SS(L,F) *such that* $1_{F}-M\in Comp\left(L,\Omega ,F\right)$ *,* $f_{pu}^{-1}(M)\in \Pi$ *.*

*(c) For every* M∈Comp(L,Ω,F) *,* $f_{pu}^{-1}(M)\in \Pi^{c}$ *.*

Proof Follows from Theorem 4.9 of [44] and Theorem 2.2. □

Lemma 2.4 *Let* (R,Π,A) *be STS,* $K\in SS(R,A)-\left\{0_{A}\right\}$ *, and* H *be soft base for* (R,Π,A) *. Then the following are equivalent:*

*(a)* K∈Comp(R,Π,A) *.*

*(b) For each* S⊆H *such that* $K\overset{˜}{\subseteq }{\overset{˜}{\cup }}_{S\in S}S$ *, there exists a finite subcollection* $S_{1}\subseteq S$ *such that* $K\overset{˜}{\subseteq }{\overset{˜}{\cup }}_{S\in S_{1}}S$ *.*

Proof (a) ⟹ (b): It is clear.

(b) ⟹ (a): Let $G\subseteq \Pi -\left\{0_{A}\right\}$ such that $K\overset{˜}{\subseteq }{\overset{˜}{\cup }}_{G\in G}G$. For every G∈G, we find $H_{G}\subseteq H$ such that ${\overset{˜}{\cup }}_{T\in H_{G}}T=G$. Let $S=\left\{T:T\in H_{G},G\in G\right\}$. Then $K\overset{˜}{\subseteq }{\overset{˜}{\cup }}_{S\in S}S$ and by (b), there exists a finite subcollection $S_{1}\subseteq S$ such that $K\overset{˜}{\subseteq }{\overset{˜}{\cup }}_{S\in S_{1}}S$. For each $S\in S_{1}$, choose $G_{S}\in G$ such that *S* $\overset{˜}{\subseteq }G_{S}$. Then $K\overset{˜}{\subseteq }{\overset{˜}{\cup }}_{S\in S_{1}}G_{S}$. Hence, K∈Comp(R,Π,A). □

Theorem 2.5 *Let* (R,Π,A) *be a STS such that A is finite and let* $K\in SS(R,A)-\left\{0_{A}\right\}$ *. Then* K∈Comp(R,Π,A) *if and only if* $K\in Comp\left(R,\Pi_{N},A\right)$ *.*

Proof *Necessity*. Suppose that K∈Comp(R,Π,A). Let H={M−C:M∈Π and C∈FSS(R,A)}. Then H is a soft base of $\left(R,\Pi_{N},A\right)$. We will apply Lemma 2.4. Let S⊆H such that $K\overset{˜}{\subseteq }{\overset{˜}{\cup }}_{S\in S}S$, say S⊆H such that $S=\{M_{\alpha }-C_{\alpha }:\alpha \in \Delta \}$ with $M_{\alpha }\in \Pi$ and $C_{\alpha }\in FSS(R,A)$ for all α∈Δ. Since K∈Comp(R,Π,A) and $K\overset{˜}{\subseteq }{\overset{˜}{\cup }}_{\alpha \in \Delta }M_{\alpha }$, then there is a finite subset $\Delta_{1}\subseteq \Delta$ such that $K\overset{˜}{\subseteq }{\overset{˜}{\cup }}_{\alpha \in \Delta_{1}}M_{\alpha }$. Put *T*= $\overset{˜}{\cup }\{C_{\alpha }:\alpha \in \Delta_{1}\}$. Then T∈FSS(R,A). For each $a_{r}\overset{˜}{\in }T$, choose $\alpha \left(a_{r}\right)\in \Delta$ such that $a_{r}\overset{˜}{\in }M_{\alpha \left(a_{r}\right)}-C_{\alpha \left(a_{r}\right)}$. Let $H_{1}=$ $\{M_{\alpha }-C_{\alpha }:\alpha \in \Delta_{1}\}\cup \left\{M_{\alpha \left(a_{r}\right)}-C_{\alpha \left(a_{r}\right)}:a_{r}\overset{˜}{\in }T\right\}$. Then $H_{1}$ is a finite subcollection of H such that $K\overset{˜}{\subseteq }{\overset{˜}{\cup }}_{H\in H_{1}}H$. Hence, $K\in Comp\left(R,\Pi_{N},A\right)$.

*Sufficiency.* It is clear. □

Corollary 2.6 *Let* (R,Π,A) *be a STS such that A is finite. Then* (R,Π,A) *is soft compact if and only if* $\left(R,\Pi_{N},A\right)$ *is soft compact.*

Theorem 2.7 *Let* $\left\{\left(R,\Pi_{a}\right):a\in A\right\}$ *be a collection of TSs such that A is finite. Then* $K\in Comp\left(R,{⊕}_{a\in A}\Pi_{a},A\right)$ *if and only if* $K(a)\in Comp\left(R,\Pi_{a}\right)$ *for all* a∈A *.*

Proof *Necessity*. Let $K\in Comp\left(R,{⊕}_{a\in A}\Pi_{a},A\right)$. Let a∈A such that K(a)≠∅, and let $B\subseteq \Pi_{a}$ such that $K(a)\subseteq \cup_{B\in B}B$. Let $H=\left\{a_{B}:B\in B\right\}\cup \left\{d_{R}:d\in A-\left\{a\right\}\right\}$. Then $H\subseteq {⊕}_{a\in A}\Pi_{a}$ such that $K\overset{˜}{\subseteq }{\overset{˜}{\cup }}_{H\in H}H$, and so, there exists a finite subcollection $H_{1}\subseteq H$ such that $K\overset{˜}{\subseteq }{\overset{˜}{\cup }}_{H\in H_{1}}H$. If $H\in H_{1}$ such that K(a)∩H(a)≠∅, then there exists B∈B such that $H=a_{B}$. Let $B_{1}=\left\{B:B\in B\text{ and }a_{B}\in H_{1}\right\}$. Then $B_{1}$ is a finite subcollection of B such that $K(a)\subseteq \cup_{B\in B_{1}}B$. Hence, $K(a)\in Comp\left(R,\Pi_{a}\right)$.

*Sufficiency.* Let $K(a)\in Comp\left(R,\Pi_{a}\right)$ for every a∈A. Let $H=\left\{a_{X}:a\in A\text{ and }X\in \Pi_{a}\right\}$. Then by Theorem 3.5 of [31], H is a soft base of $\left(R,{⊕}_{a\in A}\Pi_{a},A\right)$. We apply Lemma 2.4. Let S⊆H such that $K\overset{˜}{\subseteq }{\overset{˜}{\cup }}_{S\in S}S$. Then for every, a∈A, we have $\left\{S(a):S\in S\right\}\subseteq \Pi_{a}$ and $K(a)\overset{˜}{\subseteq }{\overset{˜}{\cup }}_{S\in S}S(a)$. So, by assumption, for every a∈A, there exists a finite subcollection $S_{a}\subseteq S$ such that $K(a)\overset{˜}{\subseteq }{\overset{˜}{\cup }}_{S\in S_{a}}S(a)$. Let $S_{0}=\left\{a_{X}:a\in A\text{ and }a_{X}\in S_{a}\right\}$. Then $S_{0}$ is a finite subcollection of S such that $K\overset{˜}{\subseteq }{\overset{˜}{\cup }}_{S\in S_{0}}S$. Hence, $K\in Comp\left(R,{⊕}_{a\in A}\Pi_{a},A\right)$. □

Corollary 2.8 *Let* (R,ς) *be a TS and let A be any finite set. Then* K∈Comp(R,τ(ς),A) *if and only if* K(a)∈Comp(R,ς) *for all* a∈A *.*

Proof For each a∈A, put $\Pi_{a}=\varsigma$. Then $\tau \left(\varsigma \right)={⊕}_{a\in A}\Pi_{a}$ and by Theorem 2.7, we get the result. □

Theorem 2.9 *Let* $\left\{\left(R,\Pi_{a}\right):a\in A\right\}$ *and* $\left\{\left(L,\Omega_{f}\right):f\in F\right\}$ *be two families of TSs such that A and F are finite. Let* p:R⟶L *and* u:A⟶F *be functions with u being bijective. Then* $f_{pu}:\left(R,{⊕}_{a\in A}\Pi_{a},A\right)⟶$ $\left(L,{⊕}_{f\in F}\Omega_{f},F\right)$ *is soft c-continuous if and only if* $p:\left(R,\Pi_{a}\right)⟶\left(L,\Omega_{u(a)}\right)$ *is c-continuous for all* a∈A *.*

Proof *Necessity*. Let $f_{pu}:\left(R,{⊕}_{a\in A}\Pi_{a},A\right)⟶$$\left(L,{⊕}_{f\in F}\Omega_{f},F\right)$ be soft *c*-continuous. Let b∈A and let $W\in \Omega_{u(b)}$ such that $L-W\in Comp\left(L,\Omega_{u(b)}\right)$. Then ${\left(u(b)\right)}_{W}\in {⊕}_{f\in F}\Omega_{f}$, and by Theorem 2.7, ${\left(u(b)\right)}_{L-W}\in Comp\left(L,{⊕}_{f\in F}\Omega_{f},F\right)$. Since u:A⟶F is injective, then $f_{pu}^{-1}({\left(u(b)\right)}_{W})=b_{p^{-1}(W)}$. Since $f_{pu}:\left(R,{⊕}_{a\in A}\Pi_{a},A\right)⟶$ $\left(L,{⊕}_{f\in F}\Omega_{f},F\right)$ is soft *c*-continuous, then $f_{pu}^{-1}({\left(u(b)\right)}_{W})=b_{p^{-1}(W)}\in {⊕}_{a\in A}\Pi_{a}$. Thus, $\left(b_{p^{-1}(W)}\right)(b)=p^{-1}(W)\in \Pi_{b}$. Hence, $p:\left(R,\Pi_{b}\right)⟶\left(L,\Omega_{u(b)}\right)$ is *c*-continuous.

*Sufficiency.* Let $p:\left(R,\Pi_{a}\right)⟶\left(L,\Omega_{u(a)}\right)$ be *c*-continuous for all a∈A. Let $G\in {⊕}_{f\in F}\Omega_{f}$ such that $1_{F}-G\in Comp\left(L,{⊕}_{f\in F}\Omega_{f},F\right)$. Then for every f∈F, $G(f)\in \Omega_{f}$ and by Theorem 2.7, $\left(1_{F}-G\right)(f)=L-G(f)\in Comp\left(L,\Omega_{f}\right)$ for all f∈F. Since u:A⟶F is bijective, then $p:\left(R,\Pi_{u^{-1}(f)}\right)⟶\left(L,\Omega_{f}\right)$ is *c*-continuous for all f∈F. Thus, $p^{-1}\left(G(f)\right)=\left(\left(f_{pu}^{-1}(G)\right)\right)(u^{-1}(f))\in \Pi_{u^{-1}(f)}$ for all f∈F. So, $\left(f_{pu}^{-1}(G)\right)(a)\in \Pi_{a}$ for all a∈A. Therefore, $f_{pu}^{-1}(G)\in {⊕}_{a\in A}\Pi_{a}$. It follows that $f_{pu}:\left(R,{⊕}_{a\in A}\Pi_{a},A\right)⟶$ $\left(L,{⊕}_{f\in F}\Omega_{f},F\right)$ is soft *c*-continuous. □

Corollary 2.10 *Let* p:(R,ς)⟶(L,δ) *and* u:T⟶F *be functions where u is a bijection between finite sets. Then* p:(R,ς)⟶(L,δ) *is c-continuous if and only if* $f_{pu}:(R,\tau \left(\varsigma \right),T)⟶(L,\tau \left(\delta \right),F)$ *is soft c-continuous.*

Proof For each t∈T and f∈F, put $\Pi_{t}=\varsigma$ and $\Omega_{f}=\delta$. Then $\tau \left(\varsigma \right)={⊕}_{t\in T}\Pi_{t}$ and $\tau \left(\delta \right)={⊕}_{f\in F}\Omega_{f}$. Theorem 2.9 ends the proof. □

Theorem 2.11 *Soft continuity implies soft c-continuity.*

Proof Let $f_{pu}:\left(R,\Pi ,B\right)⟶\left(L,\Omega ,F\right)$ be soft continuous and M∈Ω such that $1_{F}-M\in Comp\left(L,\Omega ,F\right)$. Since $f_{pu}$ is soft continuous and M∈Ω, then $f_{pu}^{-1}(M)\in \Pi$. Therefore, by Theorem 2.2 (b), $f_{pu}$ is soft *c*-continuous. □

Theorem 2.11's inverse does not have to be true in all cases:

Example 2.12 Let L=R, F={a,b}, and *ς* be the usual topology on *L*. Define p:L⟶L and u:F⟶F as follows:

$$p(l)=\left\{ \begin{array}{ccc} \frac{1}{l}\; & \text{if}\; & l\neq 0\\ \frac{1}{2}\; & \text{if}\; & l=0 \end{array}\right.,\;u(c)=c\text{ for all }c\in F.$$

It is proved in Example 2 of [35] that p:(L,ς)⟶(L,ς) is *c*-continuous but not continuous. Therefore, by Corollary 2.10 and Theorem 5.31 of [31], while $f_{pu}:(L,\tau \left(\varsigma \right),F)⟶(L,\tau \left(\varsigma \right),F)$ is soft *c*-continuous, it is not soft continuous.

The condition ‘soft Hausdorff’ in Corollary 2.3 cannot be dropped:

Example 2.13 Let S={1,2,3}, L=R, F={a,b}, ς={∅,S,{3},{2,3}}, and *δ* the topology on *L* having {(−∞,−r)∪(r,∞):r∈R} as a base. Define p:S⟶L and u:F⟶F as follows: p(s)=s for all s∈S and u(f)=f for all f∈F. To see that p:(S,ς)⟶(L,δ) is continuous, let r∈R. Then

$$p^{-1}\left(\left(-\infty ,-r\right)\cup \left(r,\infty \right)\right)=\left\{ \begin{array}{ccc} S\; & \text{if }\; & r<1\\ \left\{2,3\right\}\; & \text{if}\; & 1\leq r<2\\ \left\{3\right\}\; & \text{if}\; & 2\leq r<3\\ \emptyset \; & \text{if}\; & r\geq 3 \end{array}\right.\text{,}$$

and hence p:(S,ς)⟶(L,δ) is continuous. Thus, by Theorem 5.31 of [31] and Theorem 2.11, $f_{pu}:(S,\tau \left(\varsigma \right),F)⟶(L,\tau \left(\delta \right),F)$ is soft *c*-continuous. Since [2,3]∈Comp(L,δ), then by Corollary 2.8, $C_{\left[2,3\right]}\in Comp\left(L,\tau \left(\delta \right),F\right)$. On the other hand, $f_{pu}^{-1}\left(C_{\left[2,3\right]}\right)=C_{p^{-1}\left(\left[2,3\right]\right)}=C_{\left\{2,3\right\}}\notin {\left(\tau \left(\varsigma \right)\right)}^{c}$. Hence, $f_{pu}$ does not satisfy (c) of Corollary 2.3.

Theorem 2.14 *If* $f_{pu}:\left(R,\Pi ,A\right)⟶\left(L,\Omega ,F\right)$ *is soft c-continuous, then for any subset* X⊆R *the soft restriction* ${\left(f_{pu}\right)}_{|C_{X}}:\left(X,\Pi_{X},A\right)⟶\left(L,\Omega ,F\right)$ *is soft c-continuous.*

Proof Suppose that $f_{pu}:\left(R,\Pi ,A\right)⟶\left(L,\Omega ,F\right)$ is soft *c*-continuous and let X⊆R. Let H∈Ω such that $1_{F}-H\in Comp\left(L,\Omega ,F\right)$. Then $f_{pu}^{-1}(H)\in \Pi$ and thus, ${\left({\left(f_{pu}\right)}_{|C_{X}}\right)}^{-1}(H)=f_{pu}^{-1}(H)\overset{˜}{\cap }C_{X}\in \Pi_{X}$. Hence, ${\left(f_{pu}\right)}_{|C_{X}}:\left(X,\Pi_{X},A\right)⟶\left(L,\Omega ,F\right)$ is soft *c*-continuous. □

Theorem 2.15 *If* $f_{p_{1}u_{1}}:\left(R,\Pi ,A\right)⟶\left(L,\Omega ,F\right)$ *is soft continuous and* $f_{p_{2}u_{2}}:\left(L,\Omega ,F\right)⟶\left(N,\psi ,B\right)$ *is soft c-continuous, then* $f_{\left(p_{2}\circ p_{1}\right)\left(u_{2}\circ u_{1}\right)}:\left(R,\Pi ,A\right)⟶\left(N,\psi ,B\right)$ *is soft c-continuous.*

Proof Let M∈ψ such that $1_{B}-M\in Comp\left(N,\psi ,B\right)$. Since $f_{p_{2}u_{2}}:\left(L,\Omega ,F\right)⟶\left(N,\psi ,B\right)$ is soft *c*-continuous, then $f_{p_{2}u_{2}}^{-1}(M)\in \Omega$. Since $f_{p_{1}u_{1}}:\left(R,\Pi ,A\right)⟶\left(L,\Omega ,F\right)$ is soft continuous, then $f_{p_{1}u_{1}}^{-1}\left(f_{p_{2}u_{2}}^{-1}(M)\right)=f_{\left(p_{2}\circ p_{1}\right)\left(u_{2}\circ u_{1}\right)}^{-1}(M)\in \Pi$. It follows that $f_{\left(p_{2}\circ p_{1}\right)\left(u_{2}\circ u_{1}\right)}:\left(R,\Pi ,A\right)⟶\left(N,\psi ,B\right)$ is soft *c*-continuous. □

The following example illustrates that if $f_{p_{1}u_{1}}:\left(R,\Pi ,A\right)⟶\left(L,\Omega ,F\right)$ is soft *c*-continuous and $f_{p_{2}u_{2}}:\left(L,\Omega ,F\right)⟶\left(N,\psi ,D\right)$ is soft continuous, then $f_{\left(p_{2}\circ p_{1}\right)\left(u_{2}\circ u_{1}\right)}:\left(R,\Pi ,A\right)⟶\left(N,\psi ,D\right)$ need not be soft *c*-continuous:

Example 2.16 Let L=R, F={a,b}, and *ς* be the usual topology on *L*. Define $p_{1},p_{2}:L⟶L$ and $u_{1},u_{2}:F⟶F$ as follows:

$$p_{1}(l)=\left\{ \begin{array}{ccc} \frac{1}{l}\; & \text{if}\; & l\neq 0\\ \frac{1}{2}\; & \text{if}\; & l=0 \end{array}\right.,p_{2}(l)\text{ is the distance from }l\text{ to the nearest integer},$$

and $u_{1}$, $u_{2}$ are the identities functions. It is proved in Example 3 of [35] that $p_{1}:\left(L,\varsigma \right)⟶\left(L,\varsigma \right)$ is *c*-continuous, $p_{2}:\left(L,\varsigma \right)⟶\left(L,\varsigma \right)$ is continuous but $p_{2}\circ p_{1}:\left(L,\varsigma \right)⟶\left(L,\varsigma \right)$ is not *c*-continuous. Therefore, by Corollary 2.10 and Theorem 5.31 of [31], $f_{p_{1}u_{1}}:\left(L,\tau \left(\varsigma \right),F\right)⟶\left(L,\tau \left(\varsigma \right),F\right)$ is soft *c*-continuous and $f_{p_{2}u_{2}}:\left(L,\tau \left(\varsigma \right),F\right)⟶\left(L,\tau \left(\varsigma \right),F\right)$ is soft continuous but $f_{\left(p_{2}\circ p_{1}\right)\left(u_{2}\circ u_{1}\right)}:\left(L,\tau \left(\varsigma \right),F\right)⟶\left(L,\tau \left(\varsigma \right),F\right)$ is not soft *c*-continuous.

Theorem 2.17 *Let* $f_{p_{1}u_{1}}:\left(R,\Pi ,A\right)⟶\left(L,\Omega ,F\right)$ *be surjective and soft open. Then* $f_{p_{2}u_{2}}:\left(L,\Omega ,F\right)⟶\left(N,\psi ,B\right)$ *is soft c-continuous if* $f_{\left(p_{2}\circ p_{1}\right)\left(u_{2}\circ u_{1}\right)}:\left(R,\Pi ,A\right)⟶\left(N,\psi ,B\right)$ *is soft c-continuous.*

Proof Let M∈ψ such that $1_{B}-M\in Comp\left(N,\psi ,B\right)$. Since $f_{\left(p_{2}\circ p_{1}\right)\left(u_{2}\circ u_{1}\right)}:\left(R,\Pi ,A\right)⟶\left(N,\psi ,B\right)$ is soft *c*-continuous, then $f_{\left(p_{2}\circ p_{1}\right)\left(u_{2}\circ u_{1}\right)}^{-1}(M)=f_{p_{1}u_{1}}^{-1}\left(f_{p_{2}u_{2}}^{-1}(M)\right)\in \Pi$. Since $f_{p_{1}u_{1}}:\left(R,\Pi ,A\right)⟶\left(L,\Omega ,F\right)$ is soft open, then $f_{p_{1}u_{1}}\left(f_{p_{1}u_{1}}^{-1}\left(f_{p_{2}u_{2}}^{-1}(M)\right)\right)\in \Omega$. Since $f_{p_{1}u_{1}}$ is surjective, then $f_{p_{1}u_{1}}\left(f_{p_{1}u_{1}}^{-1}\left(f_{p_{2}u_{2}}^{-1}(M)\right)\right)=f_{p_{2}u_{2}}^{-1}(M)$. Hence, $f_{p_{2}u_{2}}:\left(L,\Omega ,F\right)⟶\left(N,\psi ,B\right)$ is soft *c*-continuous. □

Theorem 2.18 *Let* (R,Π,A) *and* (L,Ω,F) *be STSs and let* X,Y⊆R *such that* $C_{X},C_{Y}\in \Pi$ *and* $C_{X}\overset{˜}{\cup }C_{Y}=1_{A}$ *. If* $f_{pu}:\left(R,\Pi ,A\right)⟶\left(L,\Omega ,F\right)$ *is a soft function such that the soft restrictions* ${\left(f_{pu}\right)}_{|C_{X}}:\left(X,\Pi_{X},A\right)⟶\left(L,\Omega ,F\right)$ *and* ${\left(f_{pu}\right)}_{|C_{Y}}:\left(Y,\Pi_{Y},A\right)⟶\left(L,\Omega ,F\right)$ *are soft c-continuous, then* $f_{pu}:\left(R,\Pi ,A\right)⟶\left(L,\Omega ,F\right)$ *is soft c-continuous.*

Proof Let H∈Ω such that $1_{F}-H\in Comp\left(L,\Omega ,F\right)$. Then ${\left({\left(f_{pu}\right)}_{|C_{X}}\right)}^{-1}(H)\in \Pi_{X}\subseteq \Pi$ and ${\left({\left(f_{pu}\right)}_{|C_{Y}}\right)}^{-1}(H)\in \Pi_{Y}\subseteq \Pi$. Thus,

$$\begin{array}{ccc} f_{pu}^{-1}(H)\; & =\; & f_{pu}^{-1}(H)\overset{˜}{\cap }1_{A}\\ \; & =\; & f_{pu}^{-1}(H)\overset{˜}{\cap }\left(C_{X}\overset{˜}{\cup }C_{Y}\right)\\ \; & =\; & \left(f_{pu}^{-1}(H)\overset{˜}{\cap }C_{X}\right)\overset{˜}{\cup }\left(f_{pu}^{-1}(H)\overset{˜}{\cap }C_{Y}\right)\\ \; & =\; & {\left({\left(f_{pu}\right)}_{|C_{X}}\right)}^{-1}(H)\overset{˜}{\cup }{\left({\left(f_{pu}\right)}_{|C_{Y}}\right)}^{-1}(H) \end{array}$$

and hence $f_{pu}^{-1}(H)\in \Pi$. It follows that $f_{pu}:\left(R,\Pi ,A\right)⟶\left(L,\Omega ,F\right)$ is soft *c*-continuous. □

Theorem 2.19 *Let* (R,Π,A) *and* (L,Ω,F) *be STSs and let* X,Y⊆R *such that* $C_{X},C_{Y}\in \Pi^{c}$ *and* $C_{X}\overset{˜}{\cup }C_{Y}=1_{A}$ *. If* $f_{pu}:\left(R,\Pi ,A\right)⟶\left(L,\Omega ,F\right)$ *is a soft function such that the soft restrictions* ${\left(f_{pu}\right)}_{|C_{X}}:\left(X,\Pi_{X},A\right)⟶\left(L,\Omega ,F\right)$ *and* ${\left(f_{pu}\right)}_{|C_{Y}}:\left(Y,\Pi_{Y},A\right)⟶\left(L,\Omega ,F\right)$ *are soft c-continuous, then* $f_{pu}:\left(R,\Pi ,A\right)⟶\left(L,\Omega ,F\right)$ *is soft c-continuous.*

Proof Let $a_{r}\in SP(R,A)$ and let H∈Ω such that $f_{pu}\left(a_{r}\right)\overset{˜}{\in }H$ and $1_{F}-H\in Comp\left(L,\Omega ,F\right)$.

*Case 1.*r∈X∩Y. Since ${\left(f_{pu}\right)}_{|C_{X}}:\left(X,\Pi_{X},A\right)⟶\left(L,\Omega ,F\right)$ and ${\left(f_{pu}\right)}_{|C_{Y}}:\left(Y,\Pi_{Y},A\right)⟶\left(L,\Omega ,F\right)$ are soft *c*-continuous at $a_{r}$, then there exist $K_{1}\in \Pi_{X}$ and $K_{2}\in \Pi_{Y}$ such that $a_{r}\overset{˜}{\in }K_{1}\overset{˜}{\cap }K_{2}$, ${\left(f_{pu}\right)}_{|C_{X}}\left(K_{1}\right)\overset{˜}{\cup }{\left(f_{pu}\right)}_{|C_{Y}}\left(K_{2}\right)\overset{˜}{\subseteq }H$. Choose $S_{1},S_{2}\in \Pi$ such that $K_{1}=S_{1}\overset{˜}{\cap }C_{X}$ and $K_{2}=S_{2}\overset{˜}{\cap }C_{Y}$. Then

$$\begin{array}{ccc} S_{1}\overset{˜}{\cap }S_{2}\; & =\; & \left(S_{1}\overset{˜}{\cap }S_{2}\right)\overset{˜}{\cap }\left(C_{X}\overset{˜}{\cup }C_{Y}\right)\\ \; & =\; & \left(\left(S_{1}\overset{˜}{\cap }S_{2}\right)\overset{˜}{\cap }C_{X}\right)\overset{˜}{\cup }\left(\left(S_{1}\overset{˜}{\cap }S_{2}\right)\overset{˜}{\cap }C_{Y}\right)\\ \; & \overset{˜}{\subseteq }\; & \left(S_{1}\overset{˜}{\cap }C_{X}\right)\overset{˜}{\cup }\left(S_{2}\overset{˜}{\cap }C_{Y}\right)\\ \; & =\; & K_{1}\overset{˜}{\cup }K_{2} \end{array}$$

Thus, we have $a_{r}\overset{˜}{\in }S_{1}\overset{˜}{\cap }S_{2}\in \Pi$ and $f_{pu}\left(S_{1}\overset{˜}{\cap }S_{2}\right)\overset{˜}{\subseteq }f_{pu}\left(K_{1}\overset{˜}{\cup }K_{2}\right)=f_{pu}\left(K_{1}\right)\overset{˜}{\cup }f_{pu}\left(K_{2}\right)={\left(f_{pu}\right)}_{|C_{X}}\left(K_{1}\right)\overset{˜}{\cup }{\left(f_{pu}\right)}_{|C_{Y}}\left(K_{2}\right)\overset{˜}{\subseteq }H$.

*Case 2.*r∈X−Y. Since ${\left(f_{pu}\right)}_{|C_{X}}:\left(X,\Pi_{X},A\right)⟶\left(L,\Omega ,F\right)$ is soft *c*-continuous at $a_{r}$, then there exist $K\in \Pi_{X}$ such that $a_{r}\overset{˜}{\in }K$ and ${\left(f_{pu}\right)}_{|C_{X}}\left(K\right)\overset{˜}{\subseteq }H$. Choose S∈Π such that $K=S\overset{˜}{\cap }C_{X}$. Let $T=S-C_{Y}$. Then $a_{r}\overset{˜}{\in }T\in \Pi$ and $f_{pu}\left(T\right)\overset{˜}{\subseteq }f_{pu}\left(S-C_{Y}\right)=f_{pu}\left(S\overset{˜}{\cap }\left(1_{A}-C_{Y}\right)\right)\overset{˜}{\subseteq }f_{pu}\left(S\overset{˜}{\cap }C_{X}\right)=f_{pu}\left(K\right)={\left(f_{pu}\right)}_{|C_{X}}\left(K\right)\overset{˜}{\subseteq }H$.

*Case 3.*r∈Y−X. Since ${\left(f_{pu}\right)}_{|C_{Y}}:\left(Y,\Pi_{Y},A\right)⟶\left(L,\Omega ,F\right)$ is soft *c*-continuous at $a_{r}$, then there exist $K\in \Pi_{Y}$ such that $a_{r}\overset{˜}{\in }K$ and ${\left(f_{pu}\right)}_{|C_{Y}}\left(K\right)\overset{˜}{\subseteq }H$. Choose S∈Π such that $K=S\overset{˜}{\cap }C_{Y}$. Let $T=S-C_{X}$. Then $a_{r}\overset{˜}{\in }T\in \Pi$ and $f_{pu}\left(T\right)\overset{˜}{\subseteq }f_{pu}\left(S-C_{X}\right)=f_{pu}\left(S\overset{˜}{\cap }\left(1_{A}-C_{X}\right)\right)\overset{˜}{\subseteq }f_{pu}\left(S\overset{˜}{\cap }C_{Y}\right)=f_{pu}\left(K\right)={\left(f_{pu}\right)}_{|C_{Y}}\left(K\right)\overset{˜}{\subseteq }H$. □

Theorem 2.20 *Let* (R,Π,A) *and* (L,Ω,F) *be STSs and let* X,Y⊆R *such that* $C_{X}\overset{˜}{\cup }C_{Y}=1_{A}$ *. If* $f_{pu}:\left(R,\Pi ,A\right)⟶\left(L,\Omega ,F\right)$ *is a soft function such that the soft restrictions* ${\left(f_{pu}\right)}_{|C_{X}}:\left(X,\Pi_{X},A\right)⟶\left(L,\Omega ,F\right)$ *and* ${\left(f_{pu}\right)}_{|C_{Y}}:\left(Y,\Pi_{Y},A\right)⟶\left(L,\Omega ,F\right)$ *are soft c-continuous at* $a_{r}\overset{˜}{\in }C_{X}\overset{˜}{\cap }C_{Y}$ *, then* $f_{pu}:\left(R,\Pi ,A\right)⟶\left(L,\Omega ,F\right)$ *is soft c-continuous at* $a_{r}$ *.*

Proof Let H∈Ω such that $f_{pu}\left(a_{r}\right)\overset{˜}{\in }H$ and $1_{F}-H\in Comp\left(L,\Omega ,F\right)$. Since ${\left(f_{pu}\right)}_{|C_{X}}:\left(X,\Pi_{X},A\right)⟶\left(L,\Omega ,F\right)$ and ${\left(f_{pu}\right)}_{|C_{Y}}:\left(Y,\Pi_{Y},A\right)⟶\left(L,\Omega ,F\right)$ are soft *c*-continuous at $a_{r}$, then there exist K,S∈Π such that $a_{r}\overset{˜}{\in }K\overset{˜}{\cap }C_{X}\in \Pi_{X}$, $a_{r}\overset{˜}{\in }S\overset{˜}{\cap }C_{Y}\in \Pi_{Y}$, and $\left({\left(f_{pu}\right)}_{|C_{X}}\right)\left(K\overset{˜}{\cap }C_{X}\right)\overset{˜}{\cup }\left({\left(f_{pu}\right)}_{|C_{Y}}\right)\left(S\overset{˜}{\cap }C_{Y}\right)\overset{˜}{\subseteq }H$. Since

$$\begin{array}{ccc} K\overset{˜}{\cap }S\; & =\; & \left(K\overset{˜}{\cap }S\right)\overset{˜}{\cap }\left(C_{X}\overset{˜}{\cup }C_{Y}\right)\\ \; & =\; & \left(\left(K\overset{˜}{\cap }S\right)\overset{˜}{\cap }C_{X}\right)\overset{˜}{\cup }\left(\left(K\overset{˜}{\cap }S\right)\overset{˜}{\cap }C_{Y}\right)\\ \; & \overset{˜}{\subseteq }\; & \left(K\overset{˜}{\cap }C_{X}\right)\overset{˜}{\cup }\left(S\overset{˜}{\cap }C_{Y}\right)\text{,} \end{array}$$

then

$$\begin{array}{ccc} f_{pu}\left(K\overset{˜}{\cap }S\right)\; & \overset{˜}{\subseteq }\; & f_{pu}\left(\left(K\overset{˜}{\cap }C_{X}\right)\overset{˜}{\cup }\left(S\overset{˜}{\cap }C_{Y}\right)\right)\\ \; & =\; & f_{pu}\left(K\overset{˜}{\cap }C_{X}\right)\overset{˜}{\cup }f_{pu}\left(S\overset{˜}{\cap }C_{Y}\right)\\ \; & =\; & \left({\left(f_{pu}\right)}_{|C_{X}}\right)\left(K\overset{˜}{\cap }C_{X}\right)\overset{˜}{\cup }\left({\left(f_{pu}\right)}_{|C_{Y}}\right)\left(S\overset{˜}{\cap }C_{Y}\right)\\ \; & \overset{˜}{\subseteq }\; & H\text{.} \end{array}$$

It follows that $f_{pu}:\left(R,\Pi ,A\right)⟶\left(L,\Omega ,F\right)$ is soft *c*-continuous at $a_{r}$. □

Theorem 2.21 *Let* (R,Π,A) *be a STS,* (L,Ω,F) *a Hausdorff STSs, and* $f_{pu}:\left(R,\Pi ,A\right)⟶\left(L,\Omega ,F\right)$ *a soft c-continuous function. If* $C_{p(R)}$ *is a soft compact subset of* (L,Ω,F) *, then* $f_{pu}:\left(R,\Pi ,A\right)⟶\left(L,\Omega ,F\right)$ *is soft continuous.*

Proof Let $H\in \Omega^{c}$. Then $H\overset{˜}{\cap }C_{p(R)}$ is a soft closed subset of the soft compact STS $\left(p(R),\Omega_{p(R)},F\right)$, and so $H\overset{˜}{\cap }C_{p(R)}\in Comp(p(R),\Omega_{p(R)},F)$. Thus, $H\overset{˜}{\cap }C_{p(R)}\in Comp\left(L,\Omega ,F\right)$. Since (L,Ω,F) is Hausdorff, then $H\overset{˜}{\cap }C_{p(R)}\in \Omega^{c}$. Since $f_{pu}:\left(R,\Pi ,A\right)⟶\left(L,\Omega ,F\right)$ is soft *c*-continuous, then $f_{pu}^{-1}(H\overset{˜}{\cap }C_{p(R)})\in \Pi^{c}$. Hence, $f_{pu}^{-1}(H)=f_{pu}^{-1}(H\overset{˜}{\cap }C_{p(R)})\in \Pi^{c}$. Therefore, $f_{pu}:\left(R,\Pi ,A\right)⟶\left(L,\Omega ,F\right)$ is soft continuous. □

## 3 Soft almost C-continuous functions

We introduce soft almost c-continuity as a new type of soft function and investigate some of its features. We give several characterizations of it. We demonstrate with the help of examples that the class of soft almost *c*-continuous functions is precisely between the classes of soft *c*-continuous and soft almost continuous functions. Also, we show that soft almost *c*-continuity is preserved under soft restrictions. Furthermore, we investigate the conditions under which the composite of two soft almost *c*-continuous functions is soft almost *c*-continuous. In addition, we investigate the connections between this family of soft functions and their general topological analogs.

Definition 3.1 Let $f_{pu}:\left(R,\Pi ,A\right)⟶\left(L,\Omega ,F\right)$ be a soft function and let $a_{r}\in SP(R,A)$. Then

(a) $f_{pu}$ is called soft almost *c*-continuous at $a_{r}$ if for every H∈Ω such that $f_{pu}\left(a_{r}\right)\overset{˜}{\in }H$ and $1_{F}-H\in Comp\left(L,\Omega ,F\right)$, there exists M∈Π such that $a_{r}\overset{˜}{\in }M$ and $f_{pu}\left(M\right)\overset{˜}{\subseteq }Int_{\Omega }\left(Cl_{\Omega }\left(H\right)\right)$.

(b) $f_{pu}$ is called soft almost *c*-continuous if $f_{pu}$ is soft almost *c*-continuous at each soft point $a_{r}\in SP(R,A)$.

Theorem 3.2 *The following are equivalent for the soft function* $f_{pu}:\left(R,\Pi ,A\right)⟶\left(L,\Omega ,F\right)$ *:*

*(a)* $f_{pu}$ *is soft almost c-continuous.*

*(b) For every* H∈RO(L,Ω,F) *such that* $1_{F}-H\in Comp\left(L,\Omega ,F\right)$ *,* $f_{pu}^{-1}(H)\in \Pi$ *.*

*(c) For every* H∈RC(L,Ω,F)∩Comp(L,Ω,F) *,* $f_{pu}^{-1}(H)\in \Pi^{c}$ *.*

*(d) For each* $a_{r}\in SP(R,A)$ *and each* H∈RO(L,Ω,F) *such that* $f_{pu}\left(a_{r}\right)\overset{˜}{\in }H$ *and* $1_{F}-H\in Comp\left(L,\Omega ,F\right)$ *, we find* M∈Π *such that* $a_{r}\overset{˜}{\in }M$ *and* $f_{pu}\left(M\right)\overset{˜}{\subseteq }H$ *.*

*(e) For each* $a_{r}\in SP(R,A)$ *and each* H∈Ω *such that* $f_{pu}\left(a_{r}\right)\overset{˜}{\in }H$ *and* $1_{F}-H\in Comp\left(L,\Omega ,F\right)$ *,* $f_{pu}^{-1}\left(Int_{\Omega }\left(Cl_{\Omega }\left(H\right)\right)\right)\in \Pi$ *.*

Proof (a) ⟶ (b): Let H∈RO(L,Ω,F) such that $1_{F}-H\in Comp\left(L,\Omega ,F\right)$. Let $a_{r}\overset{˜}{\in }f_{pu}^{-1}(H)$. Then $f_{pu}\left(a_{r}\right)\overset{˜}{\in }H$. Since $f_{pu}$ is soft almost *c*-continuous at $a_{r}$, then there exists K∈Π such that $a_{r}\overset{˜}{\in }K$ and $f_{pu}\left(K\right)\overset{˜}{\subseteq }Int_{\Omega }\left(Cl_{\Omega }\left(H\right)\right)=H$. Thus, $a_{r}\overset{˜}{\in }K\overset{˜}{\subseteq }f_{pu}^{-1}(H)$. Hence, $f_{pu}^{-1}(H)\in \Pi$.

(b) ⟶ (c): Let H∈RC(L,Ω,F)∩Comp(L,Ω,F). Then we have $1_{F}-H\in RO\left(L,\Omega ,F\right)$ such that $1_{F}-\left(1_{F}-H\right)=H\in Comp\left(L,\Omega ,F\right)$. So, by (b), $f_{pu}^{-1}(1_{F}-H)=1_{A}-f_{pu}^{-1}(H)\in \Pi$. Hence, $f_{pu}^{-1}(H)\in \Pi^{c}$.

(c) ⟶ (d): Let $a_{r}\in SP(R,A)$ and let H∈RO(L,Ω,F) such that $f_{pu}\left(a_{r}\right)\overset{˜}{\in }H$ and $1_{F}-H\in Comp\left(L,\Omega ,F\right)$. Then we have $1_{F}-H\in RC\left(L,\Omega ,F\right)\cap Comp\left(L,\Omega ,F\right)$ and, by (c), $f_{pu}^{-1}(1_{F}-H)=1_{A}-f_{pu}^{-1}(H)\in \Pi^{c}$. Put $M=f_{pu}^{-1}(H)$. Then M∈Π such that $a_{r}\overset{˜}{\in }M$ and $f_{pu}\left(M\right)\overset{˜}{\subseteq }H$.

(d) ⟶ (e): Let $a_{r}\in SP(R,A)$ and let H∈Ω such that $f_{pu}\left(a_{r}\right)\overset{˜}{\in }H$ and $1_{F}-H\in Comp\left(L,\Omega ,F\right)$. Then we have $f_{pu}\left(a_{r}\right)\overset{˜}{\in }H\overset{˜}{\subseteq }Int_{\Omega }\left(Cl_{\Omega }\left(H\right)\right)\in RO\left(L,\Omega ,F\right)$. Since $1_{F}-Int_{\Omega }\left(Cl_{\Omega }\left(H\right)\right)$ is a soft closed subset of $1_{F}-H$ and $1_{F}-H\in Comp\left(L,\Omega ,F\right)$, then $1_{F}-Int_{\Omega }\left(Cl_{\Omega }\left(H\right)\right)\in Comp\left(L,\Omega ,F\right)$. To show that $f_{pu}^{-1}\left(Int_{\Omega }\left(Cl_{\Omega }\left(H\right)\right)\right)\in \Pi$, let $d_{s}\overset{˜}{\in }f_{pu}^{-1}\left(Int_{\Omega }\left(Cl_{\Omega }\left(H\right)\right)\right)$. Then $f_{pu}\left(d_{s}\right)\overset{˜}{\in }Int_{\Omega }\left(Cl_{\Omega }\left(H\right)\right)$. So, by (d), we find M∈Π such that $d_{s}\overset{˜}{\in }M$ and $f_{pu}\left(M\right)\overset{˜}{\subseteq }Int_{\Omega }\left(Cl_{\Omega }\left(H\right)\right)$. Thus, $d_{s}\overset{˜}{\in }M\overset{˜}{\subseteq }f_{pu}^{-1}(Int_{\Omega }\left(Cl_{\Omega }\left(H\right)\right))$. Hence, $f_{pu}^{-1}\left(Int_{\Omega }\left(Cl_{\Omega }\left(H\right)\right)\right)\in \Pi$.

(e) ⟶ (a): Let $a_{r}\in SP(R,A)$ and let H∈Ω such that $f_{pu}\left(a_{r}\right)\overset{˜}{\in }H$ and $1_{F}-H\in Comp\left(L,\Omega ,F\right)$. Put *M*= $f_{pu}^{-1}\left(Int_{\Omega }\left(Cl_{\Omega }\left(H\right)\right)\right)$. Then $a_{r}\overset{˜}{\in }f_{pu}^{-1}\left(H\right)\overset{˜}{\subseteq }f_{pu}^{-1}\left(Int_{\Omega }\left(Cl_{\Omega }\left(H\right)\right)\right)=M$ and, by (e), M∈Π. Also, $f_{pu}\left(M\right)=f_{pu}\left(f_{pu}^{-1}\left(Int_{\Omega }\left(Cl_{\Omega }\left(H\right)\right)\right)\right)\overset{˜}{\subseteq }Int_{\Omega }\left(Cl_{\Omega }\left(H\right)\right)$. □

Theorem 3.3 *Let* $\left\{\left(R,\Pi_{a}\right):a\in A\right\}$ *and* $\left\{\left(L,\Omega_{f}\right):f\in F\right\}$ *be two families of TSs such that A and F are finite sets. Let* p:R⟶L *be a function and* u:A⟶F *be a bijective function. Then* $f_{pu}:\left(R,{⊕}_{a\in A}\Pi_{a},A\right)⟶$ $\left(L,{⊕}_{f\in F}\Omega_{f},F\right)$ *is soft almost continuous if and only if* $p:\left(R,\Pi_{a}\right)⟶\left(L,\Omega_{u(a)}\right)$ *is almost continuous for every* a∈A *.*

Proof *Necessity*. Suppose that $f_{pu}:\left(R,{⊕}_{a\in A}\Pi_{a},A\right)⟶$$\left(L,{⊕}_{f\in F}\Omega_{f},F\right)$ is soft almost continuous. Let a∈A and let $W\in RO\left(L,\Omega_{u(a)}\right)$. Then by Proposition 3.28 of [45], ${\left(u(a\right))}_{W}\in RO\left(L,{⊕}_{f\in F}\Omega_{f},F\right)$. Since u:A⟶F is injective, then $f_{pu}^{-1}({\left(u(a)\right)}_{W})=a_{p^{-1}(W)}$. Since $f_{pu}:\left(R,{⊕}_{a\in A}\Pi_{a},A\right)⟶$ $\left(L,{⊕}_{f\in F}\Omega_{f},F\right)$ is soft almost continuous, then by Theorem 3.8 of [43], $f_{pu}^{-1}({\left(u(a)\right)}_{W})=a_{p^{-1}(W)}\in {⊕}_{a\in A}\Pi_{a}$. Thus, $\left(a_{p^{-1}(W)}\right)(a)=p^{-1}(W)\in \Pi_{a}$. Hence, $p:\left(R,\Pi_{a}\right)⟶\left(L,\Omega_{u(a)}\right)$ is almost continuous.

*Sufficiency.* Suppose that $p:\left(R,\Pi_{a}\right)⟶\left(L,\Omega_{u(a)}\right)$ is almost continuous for all a∈A. Let $G\in RO\left(L,{⊕}_{f\in F}\Omega_{f},F\right)$. Then by Proposition 3.28 of [45], for every f∈F, $G(f)\in RO\left(L,\Omega_{f}\right)$. Since u:A⟶F is bijective and $p:\left(R,\Pi_{u^{-1}(f)}\right)⟶\left(L,\Omega_{f}\right)$ is almost continuous for all f∈F, then $p^{-1}\left(G(f)\right)=\left(\left(f_{pu}^{-1}(G)\right)\right)(u^{-1}(f))\in \Pi_{u^{-1}(f)}$ for all f∈F. So, $\left(f_{pu}^{-1}(G)\right)(a)\in \Pi_{a}$ for all a∈A. Therefore, $f_{pu}^{-1}(G)\in {⊕}_{a\in A}\Pi_{a}$. It follows that $f_{pu}:\left(R,{⊕}_{a\in A}\Pi_{a},A\right)⟶$ $\left(L,{⊕}_{f\in F}\Omega_{f},F\right)$ is soft almost continuous. □

Corollary 3.4 *Let* p:(R,ς)⟶(L,δ) *and* u:T⟶F *be functions where u is a bijection between finite sets. Then* p:(R,ς)⟶(L,δ) *is almost continuous if and only if* $f_{pu}:(R,\tau \left(\varsigma \right),T)⟶(L,\tau \left(\delta \right),F)$ *is soft almost continuous.*

Proof For each t∈T and f∈F, put $\Pi_{t}=\varsigma$ and $\Omega_{f}=\delta$. Then $\tau \left(\varsigma \right)={⊕}_{t\in T}\Pi_{t}$ and $\tau \left(\delta \right)={⊕}_{f\in F}\Omega_{f}$. Theorem 3.3 ends the proof. □

Theorem 3.5 *Let* $\left\{\left(R,\Pi_{a}\right):a\in A\right\}$ *and* $\left\{\left(L,\Omega_{f}\right):f\in F\right\}$ *be two families of TSs such that A and F are finite sets. Let* p:R⟶L *and* u:A⟶F *be functions with u is bijective. Then* $f_{pu}:\left(R,{⊕}_{a\in A}\Pi_{a},A\right)⟶$ $\left(L,{⊕}_{f\in F}\Omega_{f},F\right)$ *is soft almost c-continuous if and only if* $p:\left(R,\Pi_{a}\right)⟶\left(L,\Omega_{u(a)}\right)$ *is almost c-continuous for every* a∈A *.*

Proof *Necessity*. Let $f_{pu}:\left(R,{⊕}_{a\in A}\Pi_{a},A\right)⟶$$\left(L,{⊕}_{f\in F}\Omega_{f},F\right)$ be soft almost *c*-continuous. Let a∈A and let $W\in RO\left(L,\Omega_{u(a)}\right)$ such that $L-W\in Comp\left(L,\Omega_{u(a)}\right)$. Then by Proposition 3.28 of [45], ${\left(u(a)\right)}_{W}\in RO\left(L,{⊕}_{f\in F}\Omega_{f},F\right)$, and by Theorem 2.7, ${\left(u(a)\right)}_{L-W}\in Comp\left(L,{⊕}_{f\in F}\Omega_{f},F\right)$. Since u:A⟶F is injective, then $f_{pu}^{-1}({\left(u(a)\right)}_{W})=a_{p^{-1}(W)}$. Since $f_{pu}:\left(R,{⊕}_{a\in A}\Pi_{a},A\right)⟶$ $\left(L,{⊕}_{f\in F}\Omega_{f},F\right)$ is soft almost *c*-continuous, then by Theorem 3.2 (b), $f_{pu}^{-1}({\left(u(a)\right)}_{W})=a_{p^{-1}(W)}\in {⊕}_{a\in A}\Pi_{a}$. Thus, $\left(a_{p^{-1}(W)}\right)(a)=p^{-1}(W)\in \Pi_{a}$. Hence, $p:\left(R,\Pi_{a}\right)⟶\left(M,\Omega_{u(a)}\right)$ is almost *c*-continuous.

*Sufficiency.* Let $p:\left(R,\Pi_{a}\right)⟶\left(L,\Omega_{u(a)}\right)$ be almost *c*-continuous for all a∈A. Let $G\in RO\left(L,{⊕}_{f\in F}\Omega_{f},F\right)$ such that $1_{F}-G\in Comp\left(L,{⊕}_{f\in F}\Omega_{f},F\right)$. Then by Proposition 3.28 of [45], for every f∈F, $G(f)\in RO\left(L,\Omega_{f}\right)$ and by Theorem 2.7, $\left(1_{F}-G\right)(f)=L-G(f)\in Comp\left(L,\Omega_{f}\right)$ for all f∈F. Since u:A⟶F is bijective, then $p:\left(R,\Pi_{u^{-1}(f)}\right)⟶\left(L,\Omega_{f}\right)$ is almost *c*-continuous for all f∈F. So, $p^{-1}\left(G(f)\right)=\left(\left(f_{pu}^{-1}(G)\right)\right)(u^{-1}(f))\in \Pi_{u^{-1}(f)}$ for all f∈F. So, $\left(f_{pu}^{-1}(G)\right)(a)\in \Pi_{a}$ for all a∈A. Therefore, $f_{pu}^{-1}(G)\in {⊕}_{a\in A}\Pi_{a}$. It follows that $f_{pu}:\left(R,{⊕}_{a\in A}\Pi_{a},A\right)⟶$ $\left(L,{⊕}_{f\in F}\Omega_{f},F\right)$ is soft almost *c*-continuous. □

Corollary 3.6 *Let* p:(R,ς)⟶(L,δ) *and* u:T⟶F *be functions where u is a bijection between finite sets. Then* p:(R,ς)⟶(L,δ) *is almost c-continuous if and only if* $f_{pu}:(R,\tau \left(\varsigma \right),T)⟶(L,\tau \left(\delta \right),F)$ *is soft almost c-continuous.*

Proof For each t∈T and f∈F, put $\Pi_{t}=\varsigma$ and $\Omega_{f}=\delta$. Then $\tau \left(\varsigma \right)={⊕}_{t\in T}\Pi_{t}$ and $\tau \left(\delta \right)={⊕}_{f\in F}\Omega_{f}$. Theorem 3.5 ends the proof. □

Theorem 3.7 *Soft c-continuity implies soft almost c-continuity.*

Proof Let $f_{pu}:\left(R,\Pi ,B\right)⟶\left(L,\Omega ,F\right)$ be soft *c*-continuous and let M∈RO(L,Ω,F)⊆Ω such that $1_{F}-M\in Comp\left(L,\Omega ,F\right)$. Since $f_{pu}$ is soft *c*-continuous, then $f_{pu}^{-1}(M)\in \Pi$. Therefore, by Theorem 3.2 (b), $f_{pu}$ is soft almost *c*-continuous. □

Theorem 3.8 *Soft almost continuous functions are soft almost c-continuous.*

Proof Let $f_{pu}:\left(R,\Pi ,A\right)⟶\left(L,\Omega ,F\right)$ be soft almost continuous and let H∈RO(L,Ω,F) such that $1_{F}-H\in Comp\left(L,\Omega ,F\right)$. As $f_{pu}$ is soft almost continuous, Theorem 3.8 of [43] implies $f_{pu}^{-1}(H)\in \Pi$. Therefore, by Theorem 3.2 (b), $f_{pu}$ is soft almost *c*-continuous. □

The converses of Theorem 3.7, Theorem 3.8 are incorrect:

Example 3.9 Let L=R, N=[0,∞), F={a,b}, *ς* be the usual topology on *L*, and *δ* be the topology on *N* having {[0,1],{1}}∪{(r,∞):r>1} as a base. Define p:L⟶N and u:F⟶F as follows: $p(l)=\left\{ \begin{array}{ccc} 2\; & \text{if}\; & l>0\\ 1\; & \text{if}\; & l=0\\ 0\; & \text{if}\; & l<0 \end{array}\right.$, and u(f)=f for all f∈F. It is proved in [36] that p:(L,ς)⟶(N,δ) is neither almost continuous nor *c*-continuous, but it is almost *c*-continuous. Therefore, by Corollary 2.10, Corollary 3.4, Corollary 3.6, $f_{pu}:(L,\tau \left(\varsigma \right),F)⟶(N,\tau \left(\delta \right),F)$ is neither soft almost continuous nor soft *c*-continuous, but it is soft almost *c*-continuous.

Theorem 3.10 *If* $f_{pu}:\left(R,\Pi ,A\right)⟶\left(L,\Omega ,F\right)$ *is soft almost c-continuous, then for any subset* X⊆R *the soft restriction* ${\left(f_{pu}\right)}_{|C_{X}}:\left(X,\Pi_{X},A\right)⟶\left(L,\Omega ,F\right)$ *is soft almost c-continuous.*

Proof Suppose that $f_{pu}:\left(R,\Pi ,A\right)⟶\left(L,\Omega ,F\right)$ is soft almost *c*-continuous and let X⊆R. Let H∈RO(L,Ω,F) such that $1_{F}-H\in Comp\left(L,\Omega ,F\right)$. Then by Theorem 3.2 (b), $f_{pu}^{-1}(H)\in \Pi$ and thus, ${\left({\left(f_{pu}\right)}_{|C_{X}}\right)}^{-1}(H)=f_{pu}^{-1}(H)\overset{˜}{\cap }C_{X}\in \Pi_{X}$. Hence, again by Theorem 3.2 (b), ${\left(f_{pu}\right)}_{|C_{X}}:\left(X,\Pi_{X},A\right)⟶\left(L,\Omega ,F\right)$ is soft almost *c*-continuous. □

Theorem 3.11 *If* $f_{p_{1}u_{1}}:\left(R,\Pi ,A\right)⟶\left(L,\Omega ,F\right)$ *is soft continuous and* $f_{p_{2}u_{2}}:\left(L,\Omega ,F\right)⟶\left(N,\psi ,D\right)$ *is soft almost c-continuous, then* $f_{\left(p_{2}\circ p_{1}\right)\left(u_{2}\circ u_{1}\right)}:\left(R,\Pi ,A\right)⟶\left(N,\psi ,D\right)$ *is soft almost c-continuous.*

Proof Let H∈RO(N,ψ,D) such that $1_{D}-H\in Comp\left(N,\psi ,D\right)$. Since $f_{p_{2}u_{2}}:\left(L,\Omega ,F\right)⟶\left(N,\psi ,D\right)$ is soft almost *c*-continuous, then $f_{p_{2}u_{2}}^{-1}(H)\in \Omega$. Since $f_{p_{1}u_{1}}:\left(R,\Pi ,A\right)⟶\left(L,\Omega ,F\right)$ is soft continuous, then $f_{p_{1}u_{1}}^{-1}\left(f_{p_{2}u_{2}}^{-1}(H)\right)=f_{\left(p_{2}\circ p_{1}\right)\left(u_{2}\circ u_{1}\right)}^{-1}(H)\in \Pi$. It follows that $f_{\left(p_{2}\circ p_{1}\right)\left(u_{2}\circ u_{1}\right)}:\left(R,\Pi ,A\right)⟶\left(N,\psi ,D\right)$ is soft almost *c*-continuous. □

Theorem 3.12 *Let* $f_{p_{1}u_{1}}:\left(R,\Pi ,A\right)⟶\left(L,\Omega ,F\right)$ *be surjective and soft open. Then* $f_{p_{2}u_{2}}:\left(L,\Omega ,F\right)⟶\left(N,\psi ,D\right)$ *is soft almost c-continuous if* $f_{\left(p_{2}\circ p_{1}\right)\left(u_{2}\circ u_{1}\right)}:\left(R,\Pi ,A\right)⟶\left(N,\psi ,D\right)$ *is soft almost c-continuous.*

Proof Let H∈RO(N,ψ,D) such that $1_{D}-H\in Comp\left(N,\psi ,D\right)$. Since $f_{\left(p_{2}\circ p_{1}\right)\left(u_{2}\circ u_{1}\right)}:\left(R,\Pi ,A\right)⟶\left(N,\psi ,D\right)$ is soft almost *c*-continuous, then $f_{\left(p_{2}\circ p_{1}\right)\left(u_{2}\circ u_{1}\right)}^{-1}(H)=f_{p_{1}u_{1}}^{-1}(f_{p_{2}u_{2}}^{-1}(H))\in \Pi$. Since $f_{p_{1}u_{1}}:\left(R,\Pi ,A\right)⟶\left(L,\Omega ,F\right)$ is soft open, then $f_{p_{1}u_{1}}(f_{p_{1}u_{1}}^{-1}(f_{p_{2}u_{2}}^{-1}(H)))\in \Omega$. Since $f_{p_{1}u_{1}}$ is surjective, then $f_{p_{1}u_{1}}(f_{p_{1}u_{1}}^{-1}(f_{p_{2}u_{2}}^{-1}(H)))=f_{p_{2}u_{2}}^{-1}(H)$. Hence, $f_{p_{2}u_{2}}:\left(L,\Omega ,F\right)⟶\left(N,\psi ,D\right)$ is soft almost *c*-continuous. □

Theorem 3.13 *Let* $f_{pu}:\left(R,\Pi ,A\right)⟶\left(L,\Omega ,F\right)$ *be a soft function and* $a_{r}\in SP(R,A)$ *. If for some* X⊆R *we have* $a_{r}\overset{˜}{\in }C_{X}\in \Pi$ *and* ${\left(f_{pu}\right)}_{|C_{X}}:\left(X,\Pi_{X},A\right)⟶\left(L,\Omega ,F\right)$ *is soft almost c-continuous at* $a_{r}$ *, then* $f_{pu}:\left(R,\Pi ,A\right)⟶\left(L,\Omega ,F\right)$ *is soft almost c-continuous at* $a_{r}$ *.*

Proof Let H∈Ω such that $f_{pu}\left(a_{r}\right)\overset{˜}{\in }H$ and $1_{F}-H\in Comp\left(L,\Omega ,F\right)$. Since ${\left(f_{pu}\right)}_{|C_{X}}:\left(X,\Pi_{X},A\right)⟶\left(L,\Omega ,F\right)$ is soft almost *c*-continuous at $a_{r}$, then there exists K∈Π such that $a_{r}\overset{˜}{\in }K\overset{˜}{\cap }C_{X}\in \Pi_{X}\subseteq \Pi$ and $\left({\left(f_{pu}\right)}_{|C_{X}}\right)\left(K\overset{˜}{\cap }C_{X}\right)=f_{pu}\left(K\overset{˜}{\cap }C_{X}\right)\overset{˜}{\subseteq }Int_{\Omega }\left(Cl_{\Omega }\left(H\right)\right)$. This shows that $f_{pu}:\left(R,\Pi ,A\right)⟶\left(L,\Omega ,F\right)$ is soft almost *c*-continuous at $a_{r}$. □

Theorem 3.14 *Let* $f_{pu}:\left(R,\Pi ,A\right)⟶\left(L,\Omega ,F\right)$ *be a soft function. If* $\left\{C_{X}:X\in X\right\}\subseteq \Pi$ *such that* ${\overset{˜}{\cup }}_{X\in X}C_{X}=1_{A}$ *and* ${\left(f_{pu}\right)}_{|C_{X}}:\left(X,\Pi_{X},A\right)⟶\left(L,\Omega ,F\right)$ *is soft almost c-continuous for every* X∈X *, then* $f_{pu}:\left(R,\Pi ,A\right)⟶\left(L,\Omega ,F\right)$ *is soft almost c-continuous.*

Proof Let $a_{r}\in SP(R,A)$. Since ${\overset{˜}{\cup }}_{X\in X}C_{X}=1_{A}$, then there exists X∈X such that $a_{r}\overset{˜}{\in }C_{X}\in \Pi$. Since by assumption, ${\left(f_{pu}\right)}_{|C_{X}}:\left(X,\Pi_{X},A\right)⟶\left(L,\Omega ,F\right)$ is soft almost *c*-continuous, then by Theorem 3.13, $f_{pu}:\left(R,\Pi ,A\right)⟶\left(L,\Omega ,F\right)$ is soft almost *c*-continuous at $a_{r}$. It follows that $f_{pu}:\left(R,\Pi ,A\right)⟶\left(L,\Omega ,F\right)$ is soft almost *c*-continuous. □

Theorem 3.15 *Let* (R,Π,A) *and* (L,Ω,F) *be STSs and let* X,Y⊆R *such that* $C_{X},C_{Y}\in \Pi^{c}$ *and* $C_{X}\overset{˜}{\cup }C_{Y}=1_{A}$ *. If* $f_{pu}:\left(R,\Pi ,A\right)⟶\left(L,\Omega ,F\right)$ *is a soft function such that the soft restrictions* ${\left(f_{pu}\right)}_{|C_{X}}:\left(X,\Pi_{X},A\right)⟶\left(L,\Omega ,F\right)$ *and* ${\left(f_{pu}\right)}_{|C_{Y}}:\left(Y,\Pi_{Y},A\right)⟶\left(L,\Omega ,F\right)$ *are soft almost c-continuous, then* $f_{pu}:\left(R,\Pi ,A\right)⟶\left(L,\Omega ,F\right)$ *is soft almost c-continuous.*

Proof Let $a_{r}\in SP(R,A)$ and let H∈Ω such that $f_{pu}\left(a_{r}\right)\overset{˜}{\in }H$ and $1_{F}-H\in Comp\left(L,\Omega ,F\right)$.

*Case 1.*r∈X∩Y. Since ${\left(f_{pu}\right)}_{|C_{X}}:\left(X,\Pi_{X},A\right)⟶\left(L,\Omega ,F\right)$ and ${\left(f_{pu}\right)}_{|C_{Y}}:\left(Y,\Pi_{Y},A\right)⟶\left(L,\Omega ,F\right)$ are soft almost *c*-continuous at $a_{r}$, then there exist $K_{1}\in \Pi_{X}$ and $K_{2}\in \Pi_{Y}$ such that $a_{r}\overset{˜}{\in }K_{1}\overset{˜}{\cap }K_{2}$ and ${\left(f_{pu}\right)}_{|C_{X}}\left(K_{1}\right)\overset{˜}{\cup }{\left(f_{pu}\right)}_{|C_{Y}}\left(K_{2}\right)\overset{˜}{\subseteq }Int_{\Omega }\left(Cl_{\Omega }\left(H\right)\right)$. Choose $S_{1},S_{2}\in \Pi$ such that $K_{1}=S_{1}\overset{˜}{\cap }C_{X}$ and $K_{2}=S_{2}\overset{˜}{\cap }C_{Y}$. Then

$$\begin{array}{ccc} S_{1}\overset{˜}{\cap }S_{2}\; & =\; & \left(S_{1}\overset{˜}{\cap }S_{2}\right)\overset{˜}{\cap }\left(C_{X}\overset{˜}{\cup }C_{Y}\right)\\ \; & =\; & \left(\left(S_{1}\overset{˜}{\cap }S_{2}\right)\overset{˜}{\cap }C_{X}\right)\overset{˜}{\cup }\left(\left(S_{1}\overset{˜}{\cap }S_{2}\right)\overset{˜}{\cap }C_{Y}\right)\\ \; & \overset{˜}{\subseteq }\; & \left(S_{1}\overset{˜}{\cap }C_{X}\right)\overset{˜}{\cup }\left(S_{2}\overset{˜}{\cap }C_{Y}\right)\\ \; & =\; & K_{1}\overset{˜}{\cup }K_{2} \end{array}$$

Thus, we have $a_{r}\overset{˜}{\in }S_{1}\overset{˜}{\cap }S_{2}\in \Pi$ and $f_{pu}\left(S_{1}\overset{˜}{\cap }S_{2}\right)\overset{˜}{\subseteq }f_{pu}\left(K_{1}\overset{˜}{\cup }K_{2}\right)=f_{pu}\left(K_{1}\right)\overset{˜}{\cup }f_{pu}\left(K_{2}\right)={\left(f_{pu}\right)}_{|C_{X}}\left(K_{1}\right)\overset{˜}{\cup }{\left(f_{pu}\right)}_{|C_{Y}}\left(K_{2}\right)\overset{˜}{\subseteq }Int_{\Omega }\left(Cl_{\Omega }\left(H\right)\right)$.

*Case 2.*r∈X−Y. Since ${\left(f_{pu}\right)}_{|C_{X}}:\left(X,\Pi_{X},A\right)⟶\left(L,\Omega ,F\right)$ is soft almost *c*-continuous at $a_{r}$, then there exist $K\in \Pi_{X}$ such that $a_{r}\overset{˜}{\in }K$ and ${\left(f_{pu}\right)}_{|C_{X}}\left(K\right)\overset{˜}{\subseteq }Int_{\Omega }\left(Cl_{\Omega }\left(H\right)\right)$. Choose S∈Π such that $K=S\overset{˜}{\cap }C_{X}$. Let $T=S-C_{Y}$. Then $a_{r}\overset{˜}{\in }T\in \Pi$ and $f_{pu}\left(T\right)\overset{˜}{\subseteq }f_{pu}\left(S-C_{Y}\right)=f_{pu}\left(S\overset{˜}{\cap }\left(1_{A}-C_{Y}\right)\right)\overset{˜}{\subseteq }f_{pu}\left(S\overset{˜}{\cap }C_{X}\right)=f_{pu}\left(K\right)={\left(f_{pu}\right)}_{|C_{X}}\left(K\right)\overset{˜}{\subseteq }Int_{\Omega }\left(Cl_{\Omega }\left(H\right)\right)$.

*Case 3.*r∈Y−X. Since ${\left(f_{pu}\right)}_{|C_{Y}}:\left(Y,\Pi_{Y},A\right)⟶\left(L,\Omega ,F\right)$ is soft almost *c*-continuous at $a_{r}$, then there exist $K\in \Pi_{Y}$ such that $a_{r}\overset{˜}{\in }K$ and ${\left(f_{pu}\right)}_{|C_{Y}}\left(K\right)\overset{˜}{\subseteq }H$. Choose S∈Π such that $K=S\overset{˜}{\cap }C_{Y}$. Let $T=S-C_{X}$. Then $a_{r}\overset{˜}{\in }T\in \Pi$ and $f_{pu}\left(T\right)\overset{˜}{\subseteq }f_{pu}\left(S-C_{X}\right)=f_{pu}\left(S\overset{˜}{\cap }\left(1_{A}-C_{X}\right)\right)\overset{˜}{\subseteq }f_{pu}\left(S\overset{˜}{\cap }C_{Y}\right)=f_{pu}\left(K\right)={\left(f_{pu}\right)}_{|C_{Y}}\left(K\right)\overset{˜}{\subseteq }Int_{\Omega }\left(Cl_{\Omega }\left(H\right)\right)$. □

Theorem 3.16 *Let* (R,Π,A) *and* (L,Ω,F) *be STSs and let* X,Y⊆R *such that* $C_{X}\overset{˜}{\cup }C_{Y}=1_{A}$ *. If* $f_{pu}:\left(R,\Pi ,A\right)⟶\left(L,\Omega ,F\right)$ *is a soft function such that the soft restrictions* ${\left(f_{pu}\right)}_{|C_{X}}:\left(X,\Pi_{X},A\right)⟶\left(L,\Omega ,F\right)$ *and* ${\left(f_{pu}\right)}_{|C_{Y}}:\left(Y,\Pi_{Y},A\right)⟶\left(L,\Omega ,F\right)$ *are soft almost c-continuous at* $a_{r}\overset{˜}{\in }C_{X}\overset{˜}{\cap }C_{Y}$ *, then* $f_{pu}:\left(R,\Pi ,A\right)⟶\left(L,\Omega ,F\right)$ *is soft almost c-continuous at* $a_{r}$ *.*

Proof Let H∈Ω such that $f_{pu}\left(a_{r}\right)\overset{˜}{\in }H$ and $1_{F}-H\in Comp\left(L,\Omega ,F\right)$. Since ${\left(f_{pu}\right)}_{|C_{X}}:\left(X,\Pi_{X},A\right)⟶\left(L,\Omega ,F\right)$ and ${\left(f_{pu}\right)}_{|C_{Y}}:\left(Y,\Pi_{Y},A\right)⟶\left(L,\Omega ,F\right)$ are soft almost *c*-continuous at $a_{r}$, then there exists K,S∈Π such that $a_{r}\overset{˜}{\in }K\overset{˜}{\cap }C_{X}\in \Pi_{X}$, $a_{r}\overset{˜}{\in }S\overset{˜}{\cap }C_{Y}\in \Pi_{Y}$, and $\left({\left(f_{pu}\right)}_{|C_{X}}\right)\left(K\overset{˜}{\cap }C_{X}\right)\overset{˜}{\cup }\left({\left(f_{pu}\right)}_{|C_{Y}}\right)\left(S\overset{˜}{\cap }C_{Y}\right)\overset{˜}{\subseteq }Int_{\Omega }\left(Cl_{\Omega }\left(H\right)\right)$. Since

$$\begin{array}{ccc} K\overset{˜}{\cap }S\; & =\; & \left(K\overset{˜}{\cap }S\right)\overset{˜}{\cap }\left(C_{X}\overset{˜}{\cup }C_{Y}\right)\\ \; & =\; & \left(\left(K\overset{˜}{\cap }S\right)\overset{˜}{\cap }C_{X}\right)\overset{˜}{\cup }\left(\left(K\overset{˜}{\cap }S\right)\overset{˜}{\cap }C_{Y}\right)\\ \; & \overset{˜}{\subseteq }\; & \left(K\overset{˜}{\cap }C_{X}\right)\overset{˜}{\cup }\left(S\overset{˜}{\cap }C_{Y}\right)\text{,} \end{array}$$

then

$$\begin{array}{ccc} f_{pu}\left(K\overset{˜}{\cap }S\right)\; & \overset{˜}{\subseteq }\; & f_{pu}\left(\left(K\overset{˜}{\cap }C_{X}\right)\overset{˜}{\cup }\left(S\overset{˜}{\cap }C_{Y}\right)\right)\\ \; & =\; & f_{pu}\left(K\overset{˜}{\cap }C_{X}\right)\overset{˜}{\cup }f_{pu}\left(S\overset{˜}{\cap }C_{Y}\right)\\ \; & =\; & \left({\left(f_{pu}\right)}_{|C_{X}}\right)\left(K\overset{˜}{\cap }C_{X}\right)\overset{˜}{\cup }\left({\left(f_{pu}\right)}_{|C_{Y}}\right)\left(S\overset{˜}{\cap }C_{Y}\right)\\ \; & \overset{˜}{\subseteq }\; & Int_{\Omega }\left(Cl_{\Omega }\left(H\right)\right)\text{.} \end{array}$$

It follows that $f_{pu}:\left(R,\Pi ,A\right)⟶\left(L,\Omega ,F\right)$ is soft almost *c*-continuous at $a_{r}$. □

Theorem 3.17 *Let* (R,Π,A) *be a STS,* (L,Ω,F) *a soft Hausdorff STSs, and* $f_{pu}:\left(R,\Pi ,A\right)⟶\left(L,\Omega ,F\right)$ *a soft almost c-continuous function. If* $C_{p(R)}$ *is a soft compact subset of* (L,Ω,F) *, then* $f_{pu}:\left(R,\Pi ,A\right)⟶\left(L,\Omega ,F\right)$ *is almost continuous.*

Proof Let $a_{r}\in SP(R,A)$ and let H∈Ω such that $f_{pu}\left(a_{r}\right)\overset{˜}{\in }H$. Then $\left(1_{F}-H\right)\overset{˜}{\cap }C_{p(R)}$ is a soft closed subset of the soft compact STS $\left(p(R),\Omega_{p(R)},F\right)$, and so $\left(1_{F}-H\right)\overset{˜}{\cap }C_{p(R)}\in Comp\left(p(R),\Omega_{p(R)},F\right)$. Thus, $\left(1_{F}-H\right)\overset{˜}{\cap }C_{p(R)}\in Comp\left(L,\Omega ,F\right)$. Since (L,Ω,F) is soft Hausdorff, then $\left(1_{F}-H\right)\overset{˜}{\cap }C_{p(R)}\in \Omega^{c}$. Since $f_{pu}:\left(R,\Pi ,A\right)⟶\left(L,\Omega ,F\right)$ is soft almost *c*-continuous, then there exists K∈Π such that $a_{r}\overset{˜}{\in }K$ and $f_{pu}\left(K\right)\overset{˜}{\subseteq }Int_{\Omega }\left(Cl_{\Omega }\left(H\right)\right)$. Therefore, $f_{pu}:\left(R,\Pi ,A\right)⟶\left(L,\Omega ,F\right)$ is soft almost continuous. □

## 4 Soft compactness

In this section, we show that for a given soft topological space (L,Ω,F), the collection of soft regular open sets with soft compact complements forms a soft base for some coarser soft topology. We demonstrate that $\left(L,\Omega^{⁎},F\right)$ are all soft compacts. Furthermore, we demonstrate that $\Omega =\Omega^{⁎}$ if (L,Ω,F) or $\left(L,\Omega^{⁎},F\right)$ is soft compact and soft Hausdorff.

Theorem 4.1 *Let* (R,Π,A) *be a STS and* $K=\left\{K\in RO\left(R,\Pi ,A\right):1_{A}-K\in Comp\left(R,\Pi ,A\right)\right\}$ *. Then* K *forms a soft base for some soft topology on R relative to A.*

Proof Since $1_{A}\in RO\left(R,\Pi ,A\right)$ and $1_{A}-1_{A}=0_{A}\in Comp\left(R,\Pi ,A\right)$, then $1_{A}\in K$. Thus, ${\overset{˜}{\cup }}_{K\in K}K=1_{A}$. Let $K_{1},K_{2}\in K$. Then $K_{1},K_{2}\in RO\left(R,\Pi ,A\right)$ and $1_{A}-K_{1},1_{A}-K_{2}\in Comp\left(R,\Pi ,A\right)$. Thus, $K_{1}\overset{˜}{\cap }K_{2}\in RO\left(R,\Pi ,A\right)$ and $1_{A}-\left(K_{1}\overset{˜}{\cap }K_{2}\right)=\left(1_{A}-K_{1}\right)\overset{˜}{\cup }\left(1_{A}-K_{2}\right)\in Comp\left(R,\Pi ,A\right)$. Hence, $K_{1}\overset{˜}{\cap }K_{2}\in K$. This shows that K forms a soft base for some soft topology on *R* relative to *A*. □

For any STS (R,Π,A), the soft topology on *R* relative to *A* having $\left\{K\in RO\left(R,\Pi ,A\right):1_{A}-K\in Comp\left(R,\Pi ,A\right)\right\}$ as a soft base will be denoted by $\Pi^{⁎}$.

Theorem 4.2 *For any STS* (R,Π,A) *,* $\left(R,\Pi^{⁎},A\right)$ *is soft compact.*

Proof Let $\left\{H:H\in H\right\}\subseteq \Pi^{⁎}$ such that ${\overset{˜}{\cup }}_{H\in H}H=1_{A}$. Let $a_{r}\in SP(R,A)$. Choose S∈H such that $a_{r}\overset{˜}{\in }S$. Since $S\in \Pi^{⁎}$, then there exists K∈RO(R,Π,A) such that $1_{A}-K\in Comp\left(R,\Pi ,A\right)$ and $a_{r}\overset{˜}{\in }K\overset{˜}{\subseteq }S$. Since $1_{A}-K\in Comp\left(R,\Pi ,A\right)$, $\left\{H:H\in H\right\}\subseteq \Pi^{⁎}\subseteq \Pi$, and $1_{A}-K\overset{˜}{\subseteq }{\overset{˜}{\cup }}_{H\in H}H$, then there exists a finite subfamily $H_{1}\subseteq H$ such that $1_{A}-K\overset{˜}{\subseteq }{\overset{˜}{\cup }}_{H\in H_{1}}H$. Let $H_{2}=\left\{S\right\}\cup H_{1}$. Then $H_{2}$ is a finite subfamily of H and ${\overset{˜}{\cup }}_{H\in H_{2}}H=1_{A}$. Hence, $\left(R,\Pi^{⁎},A\right)$ is soft compact. □

Theorem 4.3 *If* (R,Π,A) *is a soft compact Hausdorff STS, then* $\left(R,\Pi^{⁎},A\right)$ *is soft Hausdorff and* $\Pi^{⁎}=\Pi$ *.*

Proof Let $a_{r},b_{s}\in SP(R,A)$ such that $a_{r}\neq b_{s}$. Since (R,Π,A) is soft Hausdorff, then there exists M,N∈Π such that $a_{r}\overset{˜}{\in }M$, $b_{s}\overset{˜}{\in }N$, and $M\overset{˜}{\cap }N=0_{A}$. Let $M_{1}=Int_{\Pi }(Cl_{\Pi }(M))$ and $N_{1}=Int_{\Pi }(Cl_{\Pi }(N))$. Then $M_{1},N_{1}\in RO\left(R,\Pi ,A\right)$. Since (R,Π,A) is soft compact, and $1_{A}-M_{1},1_{A}-N_{1}\in \Pi^{c}$, then $1_{A}-M_{1},1_{A}-N_{1}\in Comp\left(R,\Pi ,A\right)$. Therefore, we have $M_{1},N_{1}\in \Pi^{⁎}$, $a_{r}\overset{˜}{\in }M_{1}$, $b_{s}\overset{˜}{\in }N_{1}$, and $M_{1}\overset{˜}{\cap }N_{1}=0_{A}$. This shows that $\left(R,\Pi^{⁎},A\right)$ is soft Hausdorff. Since (R,Π,A) is soft compact and soft Hausdorff, then by Theorem 3.24 of [46], (R,Π,A) is minimal soft Hausdorff. Since $\left(R,\Pi^{⁎},A\right)$ is soft Hausdorff, then Π $\subseteq \Pi^{⁎}$. Hence, $\Pi^{⁎}=\Pi$. □

Theorem 4.4 *If* (R,Π,A) *is a STS such that* $\left(R,\Pi^{⁎},A\right)$ *is soft Hausdorff, then* (R,Π,A) *is soft compact and* $\Pi^{⁎}=\Pi$ *.*

Proof We may assume that there exist $a_{r},b_{s}\in SP(R,A)$ such that $a_{r}\neq b_{s}$. Since $\left(R,\Pi^{⁎},A\right)$ is soft Hausdorff, then there exists M,N∈RO(R,Π,A)⊆Π such that $a_{r}\overset{˜}{\in }M$, $b_{s}\overset{˜}{\in }N$, $1_{A}-M$, $1_{A}-N\in Comp\left(R,\Pi ,A\right)$, and $M\overset{˜}{\cap }N=0_{A}$. Since $M\overset{˜}{\cap }N=0_{A}$, then $1_{A}=1_{A}-\left(M\overset{˜}{\cap }N\right)=\left(1_{A}-M\right)\overset{˜}{\cup }\left(1_{A}-N\right)$. Thus, $1_{A}\in Comp\left(R,\Pi ,A\right)$ and hence (R,Π,A) is soft compact. Since $\left(R,\Pi^{⁎},A\right)$ is soft Hausdorff and $\Pi^{⁎}\subseteq \Pi$, then (R,Π,A) is soft Hausdorff. Since (R,Π,A) is soft compact and soft Hausdorff, then by Theorem 3.24 of [46], (R,Π,A) is minimal soft Hausdorff. Since $\left(R,\Pi^{⁎},A\right)$ is soft Hausdorff, then Π $\subseteq \Pi^{⁎}$. Hence, $\Pi^{⁎}=\Pi$. □

Corollary 4.5 *A STS* (R,Π,A) *is soft compact Hausdorff if and only if* $\left(R,\Pi^{⁎},A\right)$ *is soft compact Hausdorff.*

Proof Follows directly from Theorem 4.3, Theorem 4.4. □

Theorem 4.6 *If* $f_{pu}:\left(R,\Pi ,A\right)⟶\left(L,\Omega ,F\right)$ *is soft almost c-continuous and* $\left(L,\Omega^{⁎},F\right)$ *is soft Hausdorff, then* $f_{pu}:\left(R,\Pi ,A\right)⟶\left(L,\Omega ,F\right)$ *is soft continuous.*

Proof Since $\left(L,\Omega^{⁎},F\right)$ is soft Hausdorff, then by Theorem 4.4, (L,Ω,F) is soft compact Hausdorff and $\Omega^{⁎}=\Omega$. To show that $f_{pu}:\left(R,\Pi ,A\right)⟶\left(L,\Omega ,F\right)$ is soft continuous, let $H\in \Omega -\left\{0_{F}\right\}$. Then $H\in \Omega^{⁎}-\left\{0_{F}\right\}$. Choose S⊆ RO(L,Ω,F) such that $H={\overset{˜}{\cup }}_{S\in S}S$ and $1_{F}-S\in Comp\left(L,\Omega ,F\right)$ for every S∈M. Since $f_{pu}:\left(R,\Pi ,A\right)⟶\left(L,\Omega ,F\right)$ is soft almost *c*-continuous, then by Theorem 3.2 (b), $f_{pu}^{-1}\left(S\right)\in \Pi$ for all S∈S. Hence, $f_{pu}^{-1}\left(H\right)=f_{pu}^{-1}=\left({\overset{˜}{\cup }}_{S\in S}S\right)={\overset{˜}{\cup }}_{S\in S}f_{pu}^{-1}\left(S\right)\in \Pi$. □

## 5 Conclusion

Since Molodtsov [1] introduced the fundamental concept of soft and Shabir and Naz [6] defined a soft topology over a family of soft sets, numerous mathematicians have extended the main notions of general topology to include soft topological spaces, such as metrization, compactness, connectedness, separation and countability axioms, paracompactness, continuity, and so forth.

In the first goal of the present paper, we extended the concepts of *c*-continuity and almost *c*-continuity to include soft topological spaces. We introduce several characterizations, relationships, and counter-examples regarding them.

In the second goal of this paper, we introduce and investigate the soft topology having soft regular open sets with soft compact complements as a new soft topology coarser than the original soft topology. We studied the relationship between our creative concepts in soft topological spaces and their topologically analogous conceptions.

Future research might look into the following topics: 1) To investigate some weaker forms of soft *c*-continuity; 2) To investigate the soft product of soft *c*-continuity and soft almost *c*-continuity.

## CRediT authorship contribution statement

**Samer Al Ghour:** conceived and designed the experiments; performed the experiments; analyzed and interpreted the data; contributed reagents, materials, analysis tools or data; wrote the paper.

## Declaration of Competing Interest

The authors declare no conflict of interest.

## Data Availability

No data was used for the research described in the article.
